# Critically Ill Pediatric Patient and SARS-CoV-2 Infection

**DOI:** 10.3390/children9040538

**Published:** 2022-04-11

**Authors:** Jozef Klučka, Eva Klabusayová, Milan Kratochvíl, Tereza Musilová, Václav Vafek, Tamara Skříšovská, Martina Kosinová, Pavla Havránková, Petr Štourač

**Affiliations:** 1 Department of Paediatric Anaesthesiology and Intensive Care Medicine, University Hospital Brno and Faculty of Medicine, Masaryk University, Kamenice 5, 625 00 Brno, Czech Republic; jozoklucka@gmail.com (J.K.); eva.klabusayova@gmail.com (E.K.); kratochvil4@akutne.cz (M.K.); musilova4@akutne.cz (T.M.); vafek4@akutne.cz (V.V.); skrisovska4@akutne.cz (T.S.); havrankova4@akutne.cz (P.H.); petr.stourac@gmail.com (P.Š.); 2 Department of Simulation Medicine, Faculty of Medicine, Masaryk University, Kamenice 5, 625 00 Brno, Czech Republic; 3 Department of Anaesthesiology and Intensive Care Medicine, The Donaustadt Clinic, Lango Bardenstraße 122, 1220 Vienna, Austria

**Keywords:** SARS-CoV-2, COVID-19, pediatric, child, management, intensive care

## Abstract

In December 2019 SARS-CoV-2 initiated a worldwide COVID-19 pandemic, which is still ongoing in 2022. Although adult elderly patients with chronic preexisting diseases had been identified as the most vulnerable group, COVID-19 has also had a significant impact on pediatric intensive care. Early in 2020, a new disease presentation, multisystemic inflammatory syndrome, was described in children. Despite the vaccination that is available for all age categories, due to its selection process, new viral mutations and highly variable vaccination rate, COVID-19 remains a significant clinical challenge in adult and pediatric intensive care in 2022.

## 1. Introduction

Since December 2019, the SARS-CoV-2 virus has had a significant impact on health-care systems all over the world, with over 3.8 million directly associated deaths being reported [1]. Due to this rapid worldwide disease spread and hospital overload, the World Health Organization (WHO) announced a global pandemic situation on 11 March 2020 [2]. The SARS-CoV-2 infection/disease–COronavirus VIrus Disease 2019 (COVID-19) was recognized and included in the International Classification of Diseases and Related Health Problems (ICD-11) under the code XN109. As in previous coronavirus epidemics (SARS-1, MERS), the primary symptoms of COVID-19 were respiratory, related to viral pneumonia and subsequent acute respiratory distress syndrome (ARDS), which was responsible for most COVID-19-related deaths. Although the disease was initially reported as being most dangerous and fatal predominantly in the elderly population (over 60 years) with major comorbidities [3], a specific pediatric-related presentation of SARS-CoV-2 infection has been described, based on the multisystemic inflammatory syndrome (Pediatric Inflammatory Multisystem Syndrome Temporally associated with COVID-19-PIMS-TS) [4]. Extraordinary research activities throughout the whole scientific community, with the aim of slowing down the pandemic as soon as possible, resulted in a unique situation in human history, as the first vaccine against SARS-CoV-2 was presented approximately one year after the first COVID-19-reported infection. Unprecedented progress has also been made in the in the treatment strategy (from Remdesivir, to corticosteroids, monoclonal antibodies and direct antivirotics against SARS-CoV-2); moreover, numerous recommendations for COVID-19 and PIMS-TS have been published since 2019. Despite the vaccination being available for all age categories, due to the selection process, as well as due to the new viral mutations and highly variable vaccination rate, COVID-19 remains a significant clinical challenge in adult and pediatric intensive care in 2022. This review aims to summarize the current evidence-based data considering the COVID-19 and PIMS-TS, and to be applicable in pediatric intensive-care settings.

## 2. SARS-CoV-2 Basic Information

Coronaviruses have been recognized as primary zoonotic RNA single-strand encapsulated viruses (with viral envelope), that can cause mild, dominantly respiratory infection in humans. The first severe acute respiratory syndrome coronavirus (SARS-CoV) epidemic in 2002 and, subsequently, the second epidemic caused by the Middle East respiratory syndrome coronavirus (MERS-CoV) in 2012, raised deep concerns regarding possible future coronavirus outbreak in the scientific communities worldwide [5]. The concerns were primarily based on its high transmissibility, severity of clinical presentation, lack of possible antiviral treatment and high lethality (15% in SARS vs. 37% in MERS) [6]. After the first published case reports/case series in December 2019, in Wuhan, China, the novel Betacoronavirus was described as SARS-CoV-2 causing atypical severe pneumonia and ARDS with high reported mortality. Its outbreak launched the world SARS-CoV-2 pandemic in 2020 [3,7,8]. SARS-CoV-2 shares 79% of its genetic information with SARS-CoV-1 and 50% with MERS-CoV [9], and most likely originated from bats or pangolins [5,10]. The virus can spread by close contact (hands, eyes, nose, mouth) and airborne transmission [10]. The virus spike (S) protein binds to the angiotensin-converting enzyme II (ACE2) receptor, expressed on the surface of several tissues (alveolar type II cells in the lungs, myocardial cells, esophageal, ileocolic cells) [2,11,12]. Rapid viral replication in the lungs can lead to pathophysiological damage on the lung tissue (bilateral diffused alveolar damage, hyaline membrane formation, desquamation of pneumocytes and fibrin deposits–primary ARDS) and/or trigger a massive immune response and create a cytokine storm, with many possible clinical features (secondary ARDS, sepsis, septic shock, multiorgan system failure (MOF), multiorgan system dysfunction (MODS)) [5,13,14,15]. Due to the rapid and widespread SARS-CoV-2 outbreak in the human population and the worldwide spread of the virus, there has been high selection potential for possible, more contagious strains (e.g., the recently dominant Omicron variant). For epidemiologic and virologic purposes, several nomenclatures have been adopted (Pango lineage, GISAID clade, Nextstrain clade) to describe so-called SARS-CoV-2 variants of concern (VOC), with increased transmissibility, virulence, severe clinical presentation and/or with decreased potential for available treatment options [16]. The most prevalent variants are listed in Table 1. The most virulent and worldwide variant, which was prevalent at the beginning of the year 2022, has been the Omicron variant. 

## 3. COVID-19 Clinical Presentation and Risk Factors

COVID-19’s clinical presentation can vary significantly, from asymptomatic infection, mild respiratory symptoms (flu-like disease), to severe or even critical infection (hypoxemia, shock, ARDS, multiple organ failure), as in Table 2 [10,17,18]. The most prevalent clinical COVID-19-related symptoms in children are cough, myalgia, fatigue, sore throat, dyspnea, hypoxemia, fever, gastrointestinal symptoms (diarrhea, nausea and vomiting, abdominal discomfort), anosmia, ageusia and multisystemic inflammation syndrome [10,17,18]. The onset of clinical symptoms can also vary (high interindividual variability, possible variability in correspondence with the viral load and viral mutation) between 1 and 14 days from transmission [10,19].

The identified risk factors for a severe course of COVID-19 are age (over 60 years and below 1 year), obesity, hypertension, pulmonary disease (e.g., COPD, asthma bronchiale), preexisting organ dysfunction, immunosuppression, oncology disease in the acute phase of treatment, vaccination status of the patient (with higher probability of severe course in those who are unvaccinated or have incomplete vaccination) [16,18]. 

## 4. Pediatric Population

Most children (up to 45%) infected with SARS-CoV-2 were asymptomatic or presented with mild upper respiratory tract infection symptoms (over 35%) according to previously published observational data [20,21]. The gastrointestinal symptoms were more prevalent in pediatric patients with COVID-19 compared to adults [22,23,24]. Another specific clinical presentation with multisystemic inflammation, shock, fever and organ failure was described in children exposed to SARS-CoV-2 as pediatric inflammatory multisystemic syndrome temporally associated with COVID-19 (PIMS) in the United Kingdom (Royal College of Pediatrics and Child Health) or as multisystemic inflammatory syndrome in children (MIS-C) in the US and Europe (Centers for Disease Control and Prevention and European Centre for Disease Control and Prevention) [25,26,27]. The pathophysiology of PIMS-TS is not yet well described. It remains unknown whether it represents a dysregulated immune response to COVID-19 infection or a new SARS-CoV-2-related childhood disease. [28,29,30]. Due to its unique clinical presentation and specific treatment algorithm, the PIMS-TS will be described in this review in more detail. 

The clinical course of COVID-19 in children, in general, is less severe than in adults. The recognized risk factor for severe complications and the need for hospital admission in children is age under 2 years [21,31,32,33,34]. Other identified risk factors for severe COVID-19 in children were obesity, chronic medical condition (e.g., diabetes mellitus, hypertension), chronic pulmonary disease, neurologic and development disorders, prematurity, age < 1 year, age < 1 month [31,32,34]. The overall reported COVID-19 mortality rate in children is below 0.2%, with in-hospital mortality between 0.1 and 8% [34,35,36]. 

## 5. Diagnostic Methods and Laboratory and Imaging Findings

The gold standard for SARS-CoV-2 detection is the viral nucleic acid findings by the real-time reverse transcriptase-polymerase chain reaction (RT-PCR) from the biospecimen obtained from the patient’s upper or lower airway mucosal samples (nasopharynx, oropharyngeal swab, tracheal aspirate, bronchoalveolar lavage) [10]. Furthermore, the viral particles can also be found in stool, urine or blood samples [37]. The nature of the PCR method can also reveal the nonviable RNA viral parts (with no invasive and replication potential). Although PCR is considered the gold standard for SARS-CoV-2 detection accepted worldwide, a positive PCR test in an asymptomatic patient does not necessarily mean active COVID-19 disease. However, due to a possible delay of up to 5 days between the initial viral exposure and positive PCR detection, one negative RT-PCR test should not exclude the possibility of COVID-19 in symptomatic patients with a high likelihood of SARS-CoV-2 infection (the testing should be repeated 1–5 days after the initial negative result) [38,39]. Rapid antigen testing from nasopharynx, oropharynx, nasal cavity or even saliva represents a relatively cheap and fast testing option, with a lower sensitivity but high specificity. Although it is possible to use antigen testing for the screening of asymptomatic patients (low time and resource requirements), their performance is best in symptomatic patients with a high viral load (early stages of COVID-19) [38]. Serologic (antibodies) testing for COVID-19 activity or to determine the immunity against SARS-CoV-2 is not recommended [38]. 

Laboratory findings in COVID-19 could reveal elevated inflammatory markers–C-reactive protein (CRP), procalcitonin, and interleukin-6 with normal or even reduced white blood cell count (leukopenia and/or lymphopenia) [10,38]. Other laboratory abnormalities that could be found in COVID-19 are elevated lactate dehydrogenase and transaminase levels, elevated D-dimers and ferritin [38]. Organ-specific laboratory findings (troponin, brain natriuretic peptide, creatinine, etc.) can be found in cases of severe or even critical course of the disease with organ failure. 

Chest X-ray or even computed tomography (CT) of the lungs in from moderate to critical COVID-19 can reveal diffuse lung tissue infiltration (pneumonia) with ground glass opacities and areas of lung consolidation (ARDS findings on CT) [38]. Lung ultrasound can reveal so-called interstitial lung syndrome with signs of lung tissue infiltration (cumulation of the vertical B-lines and diminished horizontal A lines), together with the areas of lung consolidation and “hepatization” (not aerated lung tissue) [40]. 

## 6. Initial Approach

Personnel safety issues should be considered the primary mainstay of care of a critically ill pediatric patient with COVID-19. Personal protective equipment (PPE) should be used prophylactically in all patients during the pandemic with possible COVID-19 (until proven noninfectious). The recommended PPEs are FFP2/FFP3/N95 masks, surgical mask, face shield, goggles, surgical gloves and protective gown. Ideally, the intensive care unit for COVID-19-positive patients should be separated from non-COVID-19 care. 

The initial assessment of the critically ill pediatric patient should be standardized according to local or international protocols. One of the possible approaches is the ABCDE initial approach recommended by the European Resuscitation Council (ERC) and European Paediatric Advanced Life Support taskforce (EPALS), as in Table 3 [41,42].

The primary aim of intensive care is to equalize the oxygen delivery to meet the oxygen demands, and, if possible, to normalize the oxygen requirements. The initial management using the ABCDE approach should involve the following:

### 6.1. Airway and Breathing (A+B) 

Use supplemental oxygen with nasal cannula or prongs (up to 2–3 L/min), face mask with reservoir (up to 15 L/min) or high-flow nasal cannula (up to 30 L/min and 100% O_2_) or non-invasive ventilation (NIV) to avoid hypoxemia (oxygen saturation < 90%) and/or respiratory acidosis [2]. Secure the airway if unable to protect, or invasive ventilation is needed. The cuffed tracheal tubes are recommended for all pediatric patients (improved seal, minimizing the possible viral spread). Secure the airway with rapid sequence induction algorithm by the most skilled operator on the scene with the videolaryngosope (if available) [41,43]. Use protective mechanical ventilation if possible: tidal volume ≤ 6 mL/kg, limit the plateau pressure, individualize the positive end-expiratory pressure over the lower inflection point of the compliance curve, prone position if inspired oxygen > 60%. [2,44]. Veno-venous extracorporeal membrane oxygenation (V-V ECMO) is indicated for patients with refractory hypoxemia (veno-arterial ECMO when combined with heart failure). High-frequency oscillatory ventilation (HFOV) should only be used as a rescue strategy [2,44]. 

### 6.2. Circulation (C)

The aim is to reach adequate cardiac output and meet the metabolic requirements. In patients with hypotension (according to age), 10 mL/kg balanced isotonic crystalloid in bolus form is recommended for optimizing the cardiac output. This can be repeated up to 40 mL/kg in the first hour, but the effect of treatment needs to be evaluated by echocardiography, or dynamic hemodynamic variables and indirectly by lactate level clearance (to normalize the lactate ≤ 2 mmol/L), capillary refill time (≤2 s) and mean arterial blood pressure/perfusion pressure. [2,41,45,46]. In severe hypotension, or if hypotension persists despite adequate fluid resuscitation, norepinephrine or epinephrine infusion should be initiated to normalize the above-mentioned hemodynamic parameters. The ideal route of administration is via the central vein, but the peripheral vein could be temporarily used as well. In persistent hypotension and/or high-catecholamine requirements, hydrocortisone stress dose infusion (4 mg/kg/day) and vasopressin therapy should be initiated [2,41,45,46]. In the case of ARDS, fluid-restrictive therapy is recommended. 

### 6.3. Disability (D)

The ideal neurologic state of the patient in intensive care is alert, calm, without discomfort with satisfactory analgesia, and able to cooperate and communicate with the pediatric intensive care unit (PICU) staff [47]. As defined by the Richmond agitation and sedation scale (RASS), which has recently been validated for pediatric patients, level 0 to −2 (alert to light sedation) is desirable. This could be reached with α-2 agonists (e.g., dexmedetomidine or clonidine) [47,48]. However, in case of shock and/or severe respiratory distress, the only possible initial option could be deep sedation and mechanical ventilation until organ stabilization is achieved. 

### 6.4. Exposure/Examination

Clinical examination, microbiology screening, broad-spectrum antibiotics therapy (until bacterial infection has been ruled out) and radiography examination (X-ray in all patients with possible COVID-19, CT in selected cases with severe to critical presentation) should be part of the standardized care in PICU. Echocardiography, together with cardiac enzymes and cardiology consultation, should be considered in patients with signs of shock, heart and/or multiorgan failure. 

## 7. Antiviral Treatment and Immunotherapy

### 7.1. Remdesivir

Remdesivir has been approved by the Food and Drug Administration (FDA) for COVID-19 treatment in hospitalized adults and children (≥12 years of age and ≥40 kg of weight). It could also be considered in younger children (<12 years of age and ≥3.5 kg of weight) with a high risk of disease progression, present risk factors for severe disease and in children with rapid disease progression and increasing need for oxygen [38,49]. It is indicated in patients with the need for oxygen therapy and should be administered as soon as possible (within 7 days from the first COVID-19 symptoms), but not in patients who are already on mechanical ventilation. 

### 7.2. Dexamethasone

Dexamethasone is indicated in all hospitalized children and adults with the need for oxygen therapy and/or mechanical ventilation with a dose of 0.15 mg/kg/day (maximum 6 mg/dose) for up to 10 days [38,50]. 

### 7.3. Tocilizumab

Tocilizumab is a recombinant monoclonal antibody against the interleukin-6 receptor (originally approved for rheumatoid arthritis treatment). [18,33,51]. Although mainly based on adult EBM data, it can be considered in pediatric patients with rapid disease progression and the need for oxygen therapy within 3 days of hospital admission and within 24 h after PICU admission (including patients on mechanical ventilation) [18,33]. 

Monoclonal antibodies (anti-SARS-CoV-2 drugs)–bamlanivimab + etesevimab, casirivimab + imdevimab

These monoclonal antibodies could be considered for high-risk pediatric patients that do not require oxygen therapy (≥12 years of age and ≥40 kg of weight) but, due to insufficient evidence, are only suitable for individual consideration [18,33]. However, their effectiveness against omicron variant is questionable.

Convalescent plasma, ivermectin, baricitinib, sarilumab, chloroquine/hydroxychloroquine, Isoprinosine, umifenovir are not recommended for COVID-19 treatment in pediatric patients. 

## 8. Supportive Intensive Care

Besides the standard supportive care, such as glycemia control (between 6–10 mmol/L), early enteral nutrition, and stress ulcer prophylaxis in high-risk patients [45], venous thromboembolism (VTE) prophylaxis could be considered according to the local protocols by low-molecular-weight heparin (LMWH) with an anti-Xa target between 0.3 and 0.5 IU/mL (in high-risk patients, an even higher dose may be administered, based on individual approach and consultation with hematologist) [2,33].

## 9. PIMS-TS/MIS-C

### 9.1. Definition

Early in 2020, the specific Kawasaki-like disease was first described, characterized by multisystemic inflammation and signs of organ dysfunction and/or failure presenting only in pediatric patients [4]. When the link to SARS-CoV-2 infection was identified in these patients, the new diagnosis was described as pediatric inflammatory multisystemic syndrome temporally associated with COVID-19 (PIMS-TS) in the United Kingdom (Royal College of Pediatrics and Child Health), or as multisystemic inflammatory syndrome in children (MIS-C) in US and Europe (Centers for Disease Control and Prevention and European Centre for Disease Control and Prevention) [25,26,27], as in Table 4. 

### 9.2. Clinical Presentation

Due to its multivariable clinical presentation, several PIMS-TS/MIS-C definitions have been published. Most of previously healthy pediatric patients with PIMS-TS will present with persistent fever, signs of multisystemic inflammation, and organ dysfunction (cardiac, central nervous system, renal, hepatic, coagulation), with no other explanatory diagnosis and proven or possible contact with SARS-CoV-2. Three dominant PIMS-TS clinical phenotypes were proposed for clinical consideration and further treatment decisions: 1. heart failure (left ventricular dysfunction) +/− coronary artery dilatation, 2. distributive/vasoplegic shock +/− coronary artery dilatation, 3. coronary artery dilatation and/or aneurysm formation [28]. Besides the persistent fever, respiratory infection signs, abdominal discomfort (nausea, vomiting, mitigating acute abdomen) and cardiac dysfunction (hypotension, tachycardia, laboratory results, as in Table 4.) could be found in most PIMS-TS patients. [53,54,55,56]. In the majority of patients, PIMS-TS develops between 4 and 6 weeks after COVID-19 infection and most patients have positive anti-SARS-CoV-2 antibodies on serology screening. Only a minority of patients could have PCR-positive results [4,29,38,57,58,59]. One of the possible explanations for PIMIS-TS pathophysiology could be autoantibodies-induced tissue damage [60,61]. Several case reports of a similar hyperinflammatory syndrome were also published in the adult population [62]. Due to the variety of clinical manifestations and different diagnostic criteria, with some patients remaining undiagnosed, the described prevalence of the PIMS-TS widely varies [56]. 

### 9.3. Initial Approach

The initial approach to the critically ill child with suspected PIMS-TS/MIS-C is based on the same ABCDE approach as in COVID-19 patients. The goal of the management is to restore the physiologic vital signs and, until proven otherwise, to treat the condition as septic shock (microbiology screening + antibiotics). However, PIMS-TS/MIS-C-specific treatment should not be delayed (for example, due to waiting for PCR or serology results). Wide laboratory screening (full blood count, coagulation, inflammatory markers-CPR, procalcitonin, IL-6, ferritin), together with organ-specific tests (troponin and NT-proBNP), is recommended, together with chest X-ray, 12-lead ECG, abdominal ultrasound (possible infection source) and echocardiography in all patients. The vast majority of PIMS-TS patients have elevated cardiac enzymes and abnormal echocardiography (compromised systolic function, coronary artery dilatation) and require intensive care admission [38,57]. Variable clinical presentation makes PIMS-TS difficult to diagnose, but, given the fact that the patient´s outcome is associated with the rapidity of diagnostic process, clinicians should be strongly aware of possible PIMS-TS in every febrile child with signs of organ dysfunction [38,63]. Care of PIMS-TS patients should be taken in a multidisciplinary team involving a pediatrician, intensive care physician, rheumatologist, hematologist, infectious disease physician and cardiologist. 

### 9.4. Specific Treatment

Supportive intensive care (oxygen therapy, mechanical ventilation, hemodynamic resuscitation, etc.) is the mainstay of PIMS-TS treatment [38]. Moderate to critical presentation requires specific immunomodulatory treatment, partially derived from Kawasaki disease treatment algorithm [64]. In patients with PIMS-TS and moderate to critical clinical presentation, treatment with intravenous immunoglobulins (IVIG–2 g/kg) and corticosteroids (methylprednisolone 1–2 mg/kg) is recommended [52]. This combination has been found to be superior to single IVIG treatment (shorter ICU stay, cardiac function improvement) [65]. IVIG treatment contains a significant amount of fluid load; therefore, the patient´s cardiac status should be evaluated before IVIG administration. The initial IVIG dose in patients with severe cardiac dysfunction and the high risk of fluid overload could be divided into two doses administered in 12 h intervals, or even postponed until hemodynamic stabilization (corticosteroids alone + supportive therapy) [66,67]. The second dose of IVIG could be considered (24 h after initial infusion) in case of severe clinical course and/or inadequate response to treatment [66]. Corticosteroids should be administered after sepsis/septic shock is ruled out, in case of high clinical (or laboratory) suspicion on PIMS-TS, and always in case of severe PIMS-TS cases with multiple organ dysfunction [66]. The recommended dose of 1–2 mg/kg methylprednisolone per day could be increased up to 30 mg/kg/day (1 g maximum per dose) in case of shock, multiple organ involvement and/or high vasopressor requirements [8,66]. Intravenous corticosteroids should be administered for from 3 to 4 days, followed by oral prednisone (in case of clinical improvement) [66]. For severe PIMS-TS cases with insufficient clinical response to IVIG and/or corticosteroids treatment, interleukin-1 (IL-1) receptor antagonist–Anakinra could be considered [52,66]. An alternative treatment in refractory cases could be the interleukin-6 antagonist (Tocilizumab) and tumor necrosis factor-α antagonist (Infliximab) [29,68]. Anticoagulation prophylaxis with low-molecular-weight heparin (LMWH), based on local protocol, should be considered in all PIMS-TS patients who present with at least one of the following: 5–10 times elevation of D-dimers, mild-to-moderate ventricular dysfunction, significant ECG rhythm abnormalities and coronary artery dilatation/aneurysm findings [69]. Therapeutic anticoagulation is recommended in patients with acute thrombosis, moderate-to-severe ventricular dysfunction, z-score coronary artery dilatation/aneurysm ≥ 10, D-dimer >10× elevation [69]. Due to the possible risk of coronary artery dilatation and aneurysm formation, the aspirin (3–5 mg/kg/day, up to 81 g/day) treatment is recommended in all PIMS-TS hospitalized patients without risk of bleeding (platelet count >100,000, normal coagulation tests) for at least one month [69].

### 9.5. Outcome 

The overall reported outcome of COVID-19 and PIMS-TS in children is significantly better than in adults. The reported PIMS-TS mortality reached up to 2%, but varies significantly [55,57,58,70] and roughly equals the reported mortality of COVID-19 in children 0.9–2% [71,72] with 1.3% mortality, reported on February 2022 [73]. 

## 10. Conclusions 

In the past two years of the COVID-19 worldwide pandemic, significant efforts have been made to slow down and stop the viral spread, from using simple face masks up to vaccine development. New SARS-CoV-2-associated diseases, such as PIMS-TS, have been described, and several direct antivirals (remdesivir, molnupiravir, etc.) and supportive drugs have been developed for COVID-19 and PIMS-TS. The new evidence, translated into the clinical practice, directly led to reduced morbidity and mortality associated with SARS-CoV-2. The main principle for a positive outcome is based on the early identification of patients at risk (standardized ABCDE approach) and early aggressive treatment. 

Despite the incredible progress in the care of COVID-19 and PIMS-TS patients, the battle is not over yet, as new SARS-CoV-2 variants are still being recognized in 2022 (e.g., subvariant BA.2) and the near future remains unclear, but is hopefully positive. The most effective preventive measure of a severe course of COVID-19 is vaccination, which is available for all age categories at present; therefore, among other measures, we should also focus on striving for the highest possible vaccination coverage of the population worldwide.

## Figures and Tables

**Table 1:** Currently designated variants of concern (VOCs)+ by WHO.

WHO Label	Pango Lineage	Earliest Documented Samples	Date of Designation
Alpha	B.1.1.7	United Kingdom, September 2020	18 December 2020
Beta	B.1.351	South Africa, May 2020	18 December 2020
Gamma	P.1	Brazil, November 2020	11 January 2021
Delta	B.1.617.2	India, October 2020	VOC: 11 May 2021
Omicron	B.1.1.529	Multiple countries, November 2021	VOC: 26 November 2021

Adapted from: World Health Organization [16].

**Table 2:** COVID-19 clinical presentation.

Asymptomatic infection	No clinical or radiological signs of COVID-19, positive PCR or antigen test
Mild infection	Upper respiratory tract infection symptoms—fever; flu-like symptoms—myalgia, joint pain, fever, cough, sneezing, running nose, abdominal discomfort/pain, anosmia, ageusia, no radiological signs of disease
Moderate infection	Upper and lower respiratory tract infection signs with possible wheezing, crackles, dyspnea with oxygen saturation < 94% on air, radiological signs of disease (X-ray or computed tomography) without other vital signs of deterioration
Severe infection	Dyspnea, tachypnea, hypoxemia (oxygen saturation < 94% on air) + radiological findings (in over 50% of lung tissue)
Critical infection	ARDS, encephalopathy, coagulopathy, acute renal failure, heart failure, shock, multiorgan failure + radiological findings

Adapted from: [10,18].

**Table 3:** Recommended initial approach to patients at emergency.

ABCDE Initial Approach by ERC and EPALS *		
ABCDE Approach	Aim	Action/Management
A—Airway	Airway patency, cervical spine protection if indicated	Open the mouth, bend the head (over 1 year), use airway if needed, MILS **, cervical collar or head blocks
B—Breathing	Spontaneous breathing efficacy, normoxemia, normocapnia	Pulse oximetry, oxygen, mechanical ventilation if indicated, capnography and blood gases analysis
C—Circulation	Oxygen delivery to meet the demand, blood pressure (50–95% according to age), adequate heart rate, capillary refill time ≤ 2 s, lactate ≤ 2 mmol/L	Fluid resuscitation (10 mL/kg fluid challenge), vasopressors or antihypertensives to meet target blood pressure
D—Disability	GCS ≥ 9, seizures control	Tracheal intubation and mechanical ventilation if GCS ≤ 8 and anticonvulsants
E—Exposure/Examination	Clinical examination, temperature management, normoglycemia (6–10 mmol/L)	Insulin or glucose to meet target glycemia, normothermia

* ERC (European Resuscitation Council); EPALS (European Paediatric Advanced Life Support). ** Manual in-line stabilization (of the cervical spine). ABCDE–universal initial approach to the patient, considering the importance of vital signs in alphabetical order. GCS–Glasgow coma scale.

**Table 4:** PIMS-TS and MIS-C definition.

Organization	Centers for Disease Control and Prevention USA definition	Royal College of Pediatrics and Child Health definition AND European Centre for Disease Control and Prevention definition	World Health Organization
Country	United States of America	United Kingdom and Europe	Worldwide
Syndrom/disease name	Multisystemic inflammatory syndrome in children (MIS-C)	Pediatric inflammatory multisystemic syndrome temporally associated with COVID-19 (PIMS-TS)	Multisystemic inflammatory syndrome in children (MIS-C)
Age	<21 years	All children (age not defined)	0–19 years
Clinical symptoms	Both of the following: Severe illness (hospitalized); ≥2 organ systems involved	Both of the following: Single or multiorgan dysfunction; Additional features	At least 2 of the following: Rash, conjunctivitis, and mucocutaneous inflammation; Hypotension or shock; Cardiac involvement; Coagulopathy; Acute GI symptoms
Inflammation	Laboratory evidence of inflammation including, but not limited to, 1 or more of the following: ↑CRP; ↑ESR; ↑Fibrinogen; ↑Procalcitonin; ↑D-dimer; ↑Ferritin; ↑LDH; ↑IL-6; Neutrophilia; Lymphopenia; Hypoalbuminemia	All 3 of the following: Neutrophilia; Increased CRP; lymphopenia	Elevated inflammation markers, including any of the following: ↑ESR; ↑CRP; ↑Procalcitonin
Link to SARS–CoV-2	Current or recent findings of the following: Positive by PCR; Positive by serology; Positive by antigen test; COVID-19 exposure within prior 4 weeks	Positive or negative by PCR	Evidence of COVID-19 by the following: Positive by PCR; Positive by antigen test; Positive by serology; Likely COVID-19 contact
Exclusion	No alternative diagnosis	Other infections	No obvious microbial cause

GI-gastrointestinal, CRP-c-reactive protein, ESR-erythrocyte sedimentation rate, LDH-lactate dehydrogenase, IL-6–interleukin 6. Adapted with permission from John Wiley and Sons [52].
